# Tuning Au Reactivity Beyond Canonical Targets: Ligand-Driven Au(I) Metalation of Lysine Residues in Hen Egg White Lysozyme

**DOI:** 10.1021/acs.inorgchem.6c01884

**Published:** 2026-07-08

**Authors:** Davide Piroddu, Luca Famlonga, Iogann Tolbatov, Giarita Ferraro, Lorenzo Chiaverini, Antonello Merlino, Diego La Mendola, Alessandro Marrone, Tiziano Marzo

**Affiliations:** † Department of Chemical, Physical, Mathematical and Natural Sciences, 9312 University of Sassari, Via Vienna 2, 07100 Sassari, Italy; ‡ Department of Pharmacy, University of Pisa, Via Bonanno Pisano 6, 56126 Pisa, Italy; § Department of Chemical Sciences, University of Naples Federico II, Via Cintia 21, I-80126 Napoli, Italy; ∥ Department of Pharmacy, University of Chieti-Pescara “G. D’Annunzio”, Viale Pindaro 42, 66100 Chieti, Italy

## Abstract

Au­(I) complexes are widely investigated as therapeutic agents due to their high affinity for biological nucleophiles and protein targets. Here, the reactivity of an Auranofin (AF) derivative bearing naproxen as a ligand toward hen egg white lysozyme (HEWL) was investigated by a combined crystallographic and computational approach. Notably, the first observation of lysine metalation in HEWL by an Au complex is reported. The results highlight how ligand substitution can significantly affect Au­(I) reactivity toward biomacromolecules, enabling noncanonical targeting and potentially impacting biological activity.

## Graphical abstract

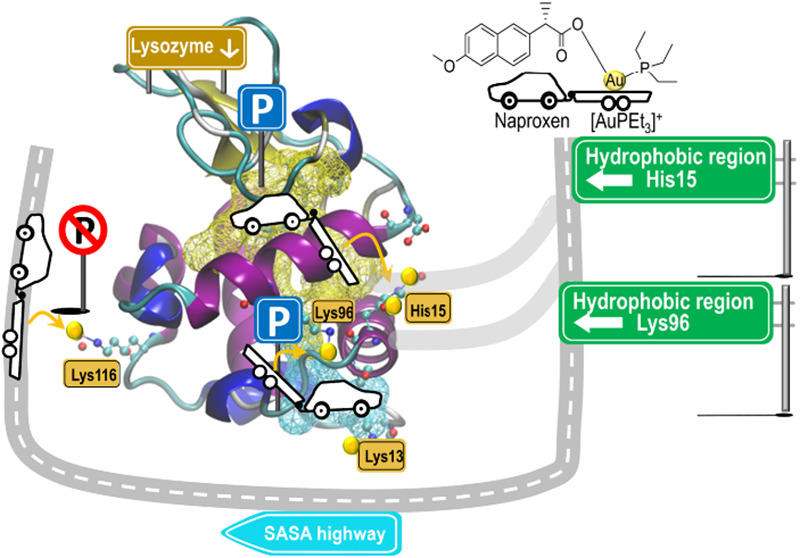

## 1 Introduction

Au­(I) complexes have attracted sustained interest in medicinal inorganic chemistry in the last decades owing to their potential pharmacological applications as anti-infective, antimicrobial, antimalarial and anticancer agents.^1–3^ The rich history of Au in medicine, dating back thousands of years, continues to stimulate new lines of research aimed at designing biologically active Au-containing compounds. Since the 1930s, several injectable Au­(I) thiolato–based drugs, including myochrysine, sanochrysine, allochrysine, and solganol, have long been used in clinical practice to treat rheumatoid arthritis, a therapeutic approach commonly known as “chrysotherapy”.^4,5^ Years later, a new antiarthritic Au­(I) complex containing a phosphine ligand, known as Auranofin (AF) (Figure 1), was introduced in clinics with the significant advantage of oral administration.^6^ Early studies indicated that AF also exhibits antimicrobial properties and anticancer activity similar to that of cisplatin,^7^ sparking great interest in the development of novel Au-based compounds for therapeutic applications.^8,9^ Unlike classical platinum drugs, whose activity is largely associated with DNA double helix binding and distortion,^10^ it has been demonstrated that Au compounds preferentially target proteins.^11–13^ Indeed, Au­(I) complexes are usually characterized by a marked thiophilicity and a strong preference for soft donor atoms. For this reason, their biological activity is primarily attributed to the formation of covalent bonds between the Au­(I) center and nucleophilic residues of proteins.^14^ An example is Thioredoxin reductase (TrxR), a selenoenzyme that plays a central role in maintaining intracellular redox homeostasis.^15^ Au­(I) compounds such as AF target the C-terminal redox-active motif of TrxR forming stable Au–S and Au–Se bonds with Cys and Sec residues (e.g., Cys497 and Sec498 in human TrxR1).^16^ Inhibition of TrxR, upon Au binding, disrupts the thioredoxin system, leading to oxidative stress, mitochondrial dysfunction, and ultimately apoptotic cell death.^17–19^

**Figure 1:**
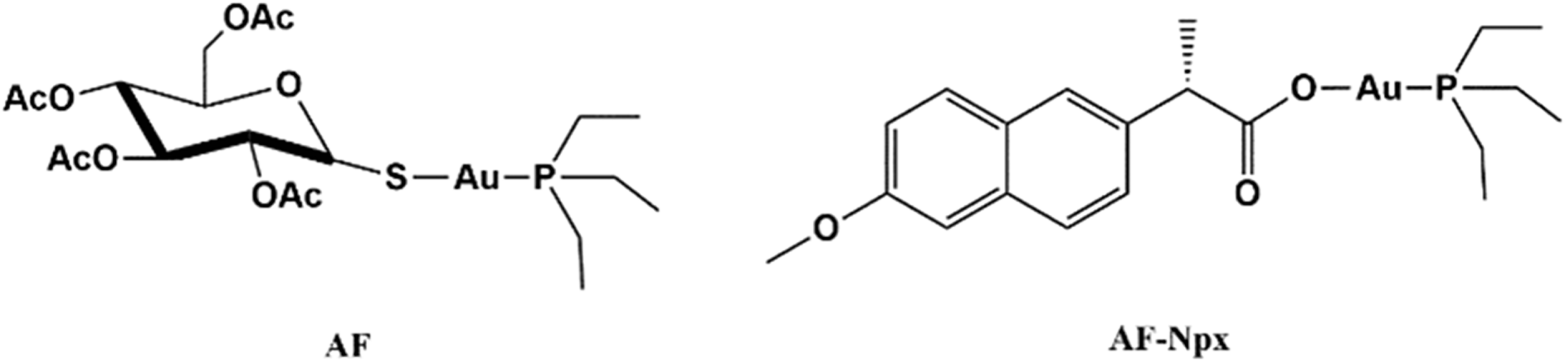
Chemical structure of Auranofin (AF) and its derivative with Naproxen (AF-Npx).

AF anticancer activity could be further improved by structural modifications aimed to modulate its reactivity, pharmacokinetic behavior, and biological profile.^20,21^ In particular, substitution of the thiosugar ligand with alternative bioactive molecules represents a promising strategy for the design of new Au complexes.^20,22,23^

The thiosugar moiety in AF primarily acts as a labile carrier ligand, stabilizing the Au­(I) center while enabling the release of the pharmacologically active [Au­(PEt_3_)]^+^ fragment under physiological conditions. Consequently, substituting this biologically inert ligand with molecules that possess intrinsic pharmacological activity may enhance the overall therapeutic effect of the complex compared with the parent drug or potentially generate synergistic biological responses.^24,25^

In this context, our group recently synthesized and studied the antimicrobial and antibiofilm activity of an AF derivative bearing naproxen (Npx) in place of the thiosugar (AF-Npx) (Figure 1), which demonstrated a 4-time lower minimum bactericidal biofilm concentration values (MBBC) for *Staphylococcus epidermidis* with respect to AF.^26^ This substitution may also lead to different biological effects that are not solely attributable to the intrinsic activity of the ligand, but also to its physicochemical properties, such as hydrophobicity and steric bulk, which can modulate the reactivity of the Au center redirecting it toward novel protein environments.

Such a design exemplifies the broader strategy of coordinating FDA-approved drugs to metal ions, a synergistic approach that can afford complexes with unique biological activities and offers a robust pathway for lab-to-market applications.^27^

Looking at future pharmacological applications, a key point for rational drug design is understanding how ligand substitution can affect the interaction of Au with proteins and its mode of binding. Indeed, metalation of plasma and cellular proteins can play a key role in determining the biological activity of metallodrugs.^28^ The interaction of Au (I) complexes with proteins generally follows a well-established pathway: activation through ligand dissociation, approach to solvent-accessible residues, and formation of coordinative bonds with nucleophilic side chains such as Cys, Met, or His.^13,17,29,30^

Upon the interaction with proteins, the original ligands coordinated to the metal center may thus not be retained in the final metal–protein adduct. Nevertheless, these ligands are often essential in guiding the metal fragment toward specific binding regions of the biomacromolecule. Especially, transient noncovalent interactions occurring in the initial stages of the recognition process can strongly influence the reaction kinetics and ultimately favor the formation of the final metalated product.^31^ Recent computational and bioinformatic studies on AF and its derivatives have highlighted that these precovalent interactions are not random, but rather a crucial preparatory step where the drug favors specific noncovalent associations with residues like Lys, effectively priming the Au center for subsequent covalent attack.^32^ Small, structurally well-defined proteins are commonly employed as model systems to elucidate the metalation process. In this regard, hen egg white lysozyme (HEWL) has been widely employed because of its relatively small size, high solubility, structural stability, and low cost. In addition, it is well suited for ESI-MS analyses and easily crystallizes under a broad range of experimental conditions. HEWL crystals are typically resistant to soaking procedures. This is particularly advantageous for structural investigations of metal–protein interactions.^33^

In the present work, we investigated the reactivity of AF-Npx toward HEWL to assess how replacement of the thiosugar with a hydrophobic carboxylate ligand influences Au release and protein metalation. Notably, the presence of Npx leads to a binding pattern that differs from that typically observed for Au­(I) complexes with HEWL, with metalation occurring at residues that are not commonly targeted (Lys116, Lys13 and Lys96). To the best of our knowledge, this represents the first observation of lysine metalation in HEWL by a Au complex. Interaction of Au to Lys side chains was previously reported in the structure of the adduct formed by Au­(NHC)Cl (with NHC = 1-butyl-3-methyl-imidazole-2-ylidene) with the model protein thaumatin.^34^

Combining X-ray crystallography with computational analysis, we elucidate how both structural and electronic effects introduced by ligand substitution can modulate the reactivity of the Au­(I) center and redirect its binding within HEWL. Overall, this approach provides insight into how ligand design can be leveraged to tune the protein-targeting properties of Au­(I) metallodrugs.

## 2 Results

### 2.1 X-ray Crystallographic Studies

To study the interaction of AF-Npx with HEWL from the structural point of view, X-ray crystallographic studies were carried out. To gain more information on the possible binding of Au to the model protein, the adducts HEWL:AF-Npx were obtained in two different experimental conditions: 2.0 M sodium formate, 0.1 M Hepes buffer pH 7.5 (Structure **1**) (Figure 2) and 14% PEG 8K, 0.1 M sodium citrate, 0.1 M sodium cacodylate buffer pH 6.5 (Structure **2**) (Figure 3). Crystals of Structure **1**, obtained after 6 days of soaking, and Structure **2**, obtained after 1 day of soaking, diffract X-rays at 1.54 and 1.44 Å resolution, respectively (Table S1). The structures were refined up to R-factor/R_free_ of 0.208/0.250 and 0.181/0.200, respectively. The overall structures of the protein in the adducts with Au are very similar to that of the complex-free protein; the root-mean-square deviation of carbon α atoms (r.m.s.d.) between the structure of each HEWL-Au adduct and that of metal-free HEWL used as the starting model (PDB code193L)^35^ is 0.18 and 0.25 Å, respectively. Inspection of Fourier difference and anomalous difference electron density maps (Figures 2 and 3) clearly indicates the presence of up to four Au binding sites. Upon reaction with the protein, AF-Npx undergoes degradation, releasing Au-containing fragments which coordinate to the side chains of different protein residues. In both structures, the electron density map around the metal centers is not well-defined and did not allow to complete the modeling of Au coordination sphere. The first Au binding site was found close to His15, where a Au atom, with partial occupancy (0.70 in Structure **1**, 0.50 in Structure **2**), interacts with the ND1 atom and with a water molecule. A second Au atom is bound to NE2 atom of His15 in Structure **2** (occupancy= 0.20). The linear geometry around the metal centers suggests that, at these sites, Au is in the oxidation state +1 (Figures 2B and 3B). ND1 and NE2 atoms of His15 are common metalation sites in HEWL, not only for Au-based compounds but also for platinum complexes.^36–42^ In the present structures, beyond the common His15, other binding sites, quite uncommon for a Au compound, were detected. Indeed, inspection of electron density maps shows the presence of Au-containing fragments coordinated to the NZ atoms of Lys96 and Lys116 in both structures of HEWL:AF-Npx adduct (Figures 2C,D and 3D,E), and to the NZ atom of Lys13 in Structure **2** (Figure 3C). Although the anomalous signal unequivocally identifies Au at these sites, the electron density surrounding the metal centers is insufficient to confidently model the complete coordination sphere. In these sites, distances between NZ and Au are within the range 2.17–2.50 Å and occupancy is within the range 0.20–0.50. These Au binding sites have never been observed in the adducts of HEWL with Au compounds.

**Figure 2:**
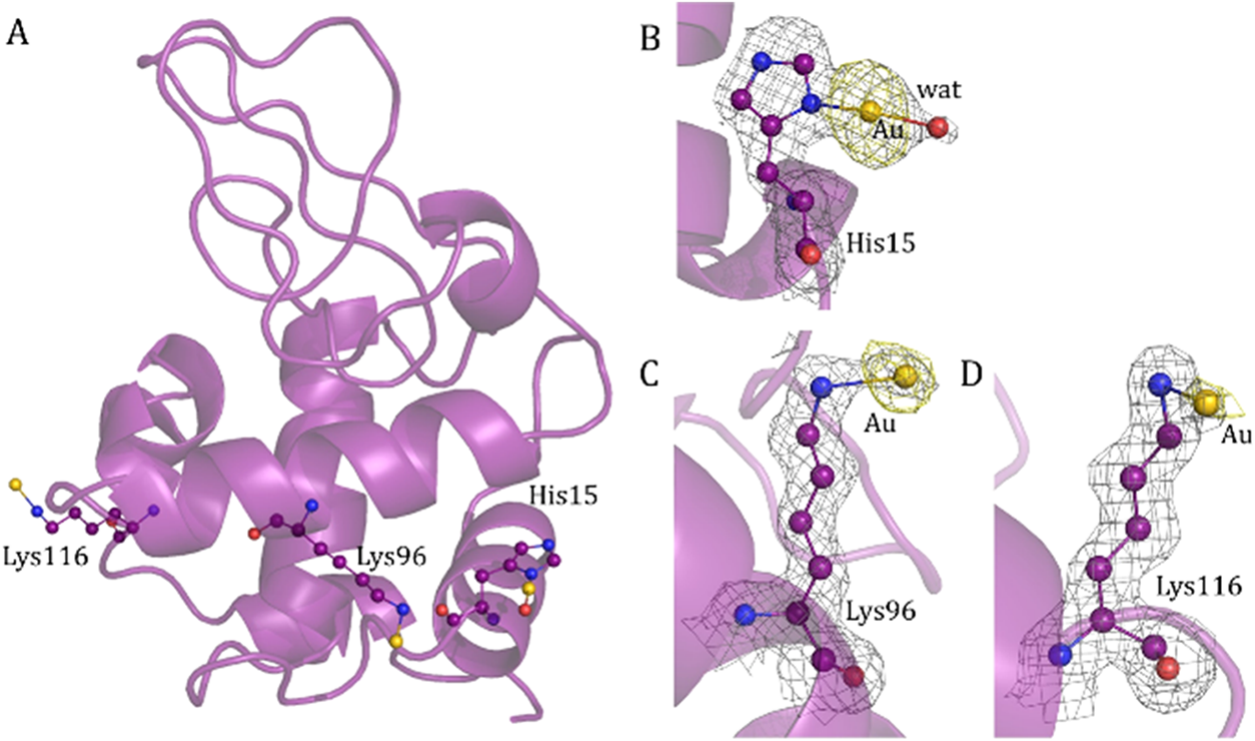
Cartoon representation of Structure **1** (PDB code:29OH, panel A). AF-Npx binding sites are shown in ball and sticks. In panels B–D, magnification of each AF-Npx binding site is reported. 2Fo-Fc electron density map is contoured at 1.0 σ level and colored in dark gray. Anomalous difference electron density map is contoured at 3.0 σ level and colored in yellow.

**Figure 3:**
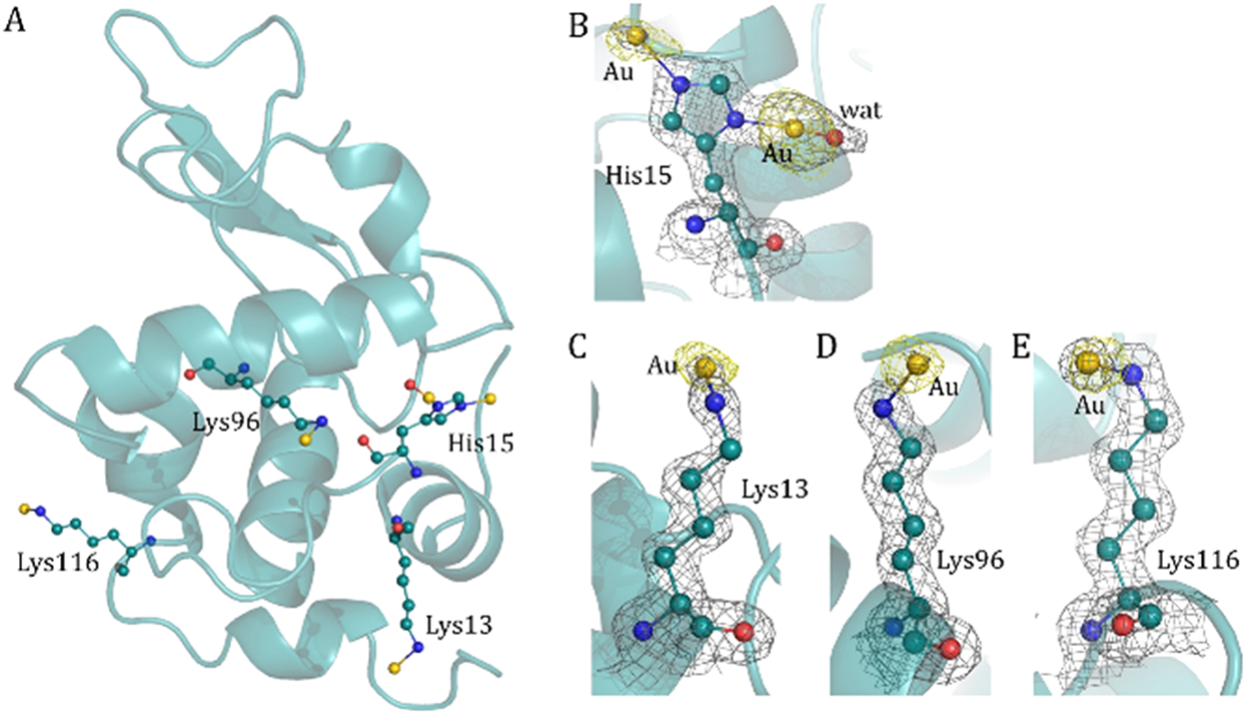
Cartoon representation of Structure **2** (PDB code:29OL, panel A). AF-Npx binding sites are shown in ball and sticks. In panels B–E, magnification of each AF-Npx binding site is reported. 2Fo-Fc electron density map is contoured at 1.0 σ level and colored in dark gray. Anomalous difference electron density map is contoured at 3.0 σ level and colored yellow.

Analyzing the X-ray structures formed upon reaction of HEWL with Au compounds deposited in the PDB (Protein Data Bank, Table S2), preferential binding sites for Au on HEWL structure can be identified. As expected, His15 is the most observed Au binding site. It has been shown, for example, that reaction of medicinal Au compounds, like AuSac_2_, Auoxo6, or Au_2_phen, with HEWL leads to the formation of the same type of metal–protein adduct bearing a Au­(I) ion tightly anchored to His15 with a chloride ion or a water molecule as second ligand, even in the case of Au­(III) complexes which act as pro-drugs.^43^

Additional Au binding sites are found close to Asn93 and Gln121. The structure of the adduct that HEWL forms with Aubipy^c^, for example, revealed the presence of a Au ion bound to the side chain of Gln121.^43^

Since Au­(I) has a preference for soft ligands such as sulfur and phosphorus, Cys or Met residues are expected as preferential secondary binding sites.^44^ However, it seems that Au­(I) centers bind tightly to HEWL, most likely to oxygen or nitrogen donors.^45^ Only in one case,^46^ a Au­(I) ion bound to the SD atom of the zero-solvent accessible Met105 side chain, which is located in the protein hydrophobic box, is observed.

So, data collected up to now suggest that Au coordination to specific protein residues can be directed modulating the experimental conditions or designing ligands able to “guide” the metal center to specific protein regions. Indeed, coupling the information provided from literature and those obtained from the crystallographic results, we hypothesized that Npx can modulate AF-Npx binding to HEWL, producing unusual adducts.

### 2.2 Computational Analysis

To confirm our hypothesis and explain our crystallographic results, computational analyses were carried out.

The analysis of the solvent accessible surface area (SASA) data for the metalated residues in our structures reveals a difference in the observed Au binding sites in terms of solvent accessibility. To avoid the masking effect of the metal, SASA values were calculated using the metal-free HEWL (PDB 193L) as a baseline (Table 1).

**Table 1:** SASA and Apolar Character of Metalated Lys and His Residues in the Unbound HEWL (apo) and HEWL:AF-Npx Adducts (Structures **1** and **2**)

Structure	Residue	Total SASA (Å^2^)	Apolar SASA (Å^2^)	Exposure (%)
**apo**		35.7	11.3	18.3
**Structure 1**	His15	25.3	6.8	11.9
**Structure 2**		44.9	3.8	21.8
**apo**		39.0	15.1	17.1
**Structure 1**	Lys96	32.2	17.1	13.7
**Structure 2**		35.2	11.3	16.8
**apo**		107.2	52.8	46.3
**Structure 1**	Lys116	85.2	30.8	41.4
**Structure 2**		110.4	49.1	48.4
**apo**		67.7	30.2	31.1
**Structure 2**	Lys13	85.3	37.7	39.5

The Au binding sites observed in our structures can be classified into two categories. Based on their fractional exposure (Table 1), His15 and the newly identified Lys96 are moderately solvent-exposed. Their total SASA values in the apo form are 35.7 and 39.0 Å^2^, respectively. In contrast, residues Lys116 and Lys13 are highly exposed. These residues exhibit high accessibility, with SASA in the apo form of 107.2 and 67.7 Å^2^. This high exposure suggests that, for these two sites, the kinetic accessibility of the residue to the Au­(I) species in solution is a primary factor in their metalation. On the contrary, the values for the metalated structures **1** and **2** show the direct impact of coordination. In Structure **1**, a decrease in total SASA (e.g., from 35.7 to 25.3 Å^2^ for His15) is observed, consistent with the physical masking of the residue by the Au-containing fragment. Conversely, in Structure **2**, an increase in SASA is noted for His15 and Lys13 compared to the apo-form, suggesting that the interaction with the Au-fragment may induce localized structural rearrangements. While these SASA values are derived from a static structural model and do not account for the intrinsic conformational flexibility of the protein, the observed accessibility gradient remains a robust descriptor for the ligand-driven anchor effect, capturing the relative exposure of target residues prior to and upon metalation. The evaluation of apolarity and hydrophobicity based on the apolar SASA provides additional information that could be useful to explain the observed Au binding sites. The highly exposed residues, Lys116 and Lys13, possess a high degree of apolar exposure in the apo form (52.8 and 30.2 Å^2^). This suggests the Au­(I) complex binds to these surface sites, that are both accessible and partially hydrophobic, thanks to Npx, that acts as a hydrophobic anchor to guide the pro-drug toward HEWL nonpolar regions. Interestingly, in the metalated adducts, the apolar SASA of these residues remains high (e.g., 49.1 Å^2^ for Lys116 in Structure **2**), confirming that the hydrophobic nature of the binding site is maintained or even reinforced upon the binding. Conversely, the moderately exposed His15 and Lys96 exhibit a low apolar contribution in the apo form (11.3 and 15.1 Å^2^). In the case of these residues, it is possible that Npx guidance must rely on the hydrophobic environment of the pocket. This suggests that AF-Npx targets pockets where the nonpolar Npx group is thermodynamically stabilized, forcing the Au­(I) center in proximity with the available nucleophile (Lys or His). Indeed, Lys96 and His15 are located close to two hydrophobic clusters (Figure 4): Cluster 1, comprising residues Leu8, Leu17, Ile55, Leu56, Ile58, Ile78, Leu83, Ile88, and Ile98, and Cluster 2, formed by Leu25, Val29, Ile124, and Leu129.

**Figure 4:**
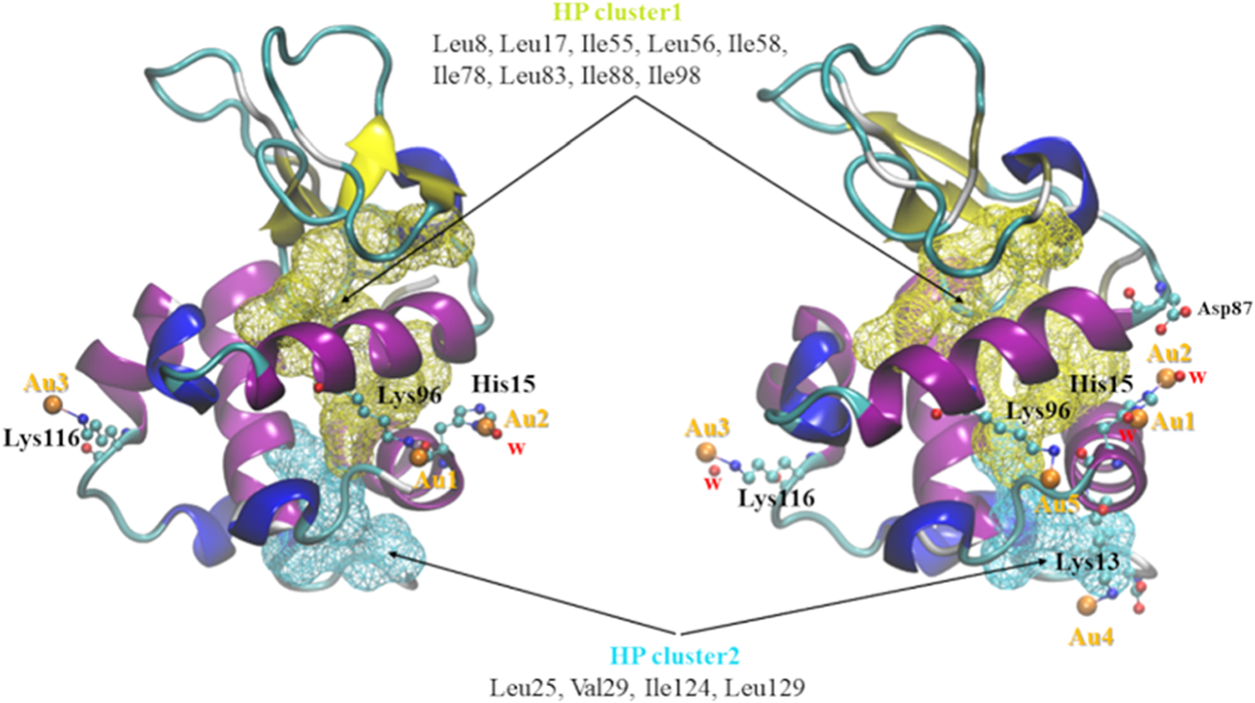
Visual mapping of hydrophobic clusters close to Au­(I) binding sites in the HEWL:AF-Npx adduct.

This suggests that the mechanism that leads to the binding of Au to His15 and Lys96 is distinct from that leading to Au anchoring to Lys13 and Lys116: moderately accessible sites require thermodynamic guidance via the hydrophobic anchor by Npx while highly exposed residues are metalated through a simple kinetic pathway facilitated by their high solvent exposure.

To complement the structural analysis, we investigated the intrinsic reactivity of the AF-Npx complex compared to its parent compound, AF, through conceptual DFT descriptors (Table 2).

**Table 2:** Conceptual DFT Descriptors for AF and AF-Npx

Complex	Chemical potential (μ)	Hardness (η)	Softness (s)	Electrophilicity (ω)
**AF**	–0.128	0.172	2.908	0.048
**AF-Npx**	–0.133	0.151	3.308	0.058

Data reveal that the substitution of the thiosugar in AF with the Npx moiety in AF-Npx significantly alters the electronic profile of the Au­(I) center. Specifically, AF-Npx exhibits a lower chemical potential (**μ**) and a higher global electrophilicity (**ω**) compared to AF.

This indicates that the Au­(I) center in AF-Npx is more electron-deficient and inherently more electrophilic, making it more reactive toward protein nucleophiles. Furthermore, the simultaneous decrease in global hardness (**η**) and corresponding increase in global softness (s) for AF-Npx reveal a subtle interplay within these conceptual DFT descriptors. While an increase in global softness traditionally signifies a heightened qualitative selectivity toward soft donors (like Cys or thiolates), according to the HSAB principle, the noncanonical reactivity observed here is explicitly dictated by the sharp elevation in global electrophilicity (from 0.048 to 0.058) and the concurrent drop in electronic chemical potential (μ). This substantial electronic activation yields a powerful frontier orbital driving force that effectively overrides the intrinsic HSAB mismatch, enabling the Au­(I) fragment to bypass its conventional selectivities and metalate the harder Lys residues.

In synthesis, the hydrophobic Npx moiety provides the spatial guidance through the anchor effect, while the altered electronic structure of AF-Npx provides the necessary electronic activation to form stable bonds with Lys nitrogens. These descriptors numerically confirm our hypothesis that the Au­(I) center in AF-Npx is more electrophilic and inherently more reactive than that of standard AF.

The observed metalation pattern can be rationalized through a two-step recognition process. First, the hydrophobic Npx moiety promotes transient noncovalent association with specific protein regions, increasing the local concentration of the Au-containing fragment near potential nucleophilic residues. Subsequently, electron donation from the nitrogen lone pair of Lys or His to the electrophilic Au­(I) center enables formation of a coordinate Au–N bond, ultimately yielding the experimentally observed metalated adducts.

To further quantify the competition between the identified binding sites, we calculated the nucleophilic Fukui indices (*f* ^–^) for the nitrogen atoms of His and Lys (Table 3). The data show that the unprotonated Lys NZ atom possesses a high intrinsic nucleophilicity (*f* ^–^ = −0.515), which numerically exceeds that of the His imidazole nitrogens.

**Table 3:** Nucleophilic Fukui Indices (*f* ^–^) for the Target Nitrogen Atoms in His and Lys Residues

Residue	Atom	*f* ^–^
His	*ND* **1**	–0.089
His	*NE* **2**	–0.073
Lys	*NZ*	–0.515

While His is classically preferred due to the HSAB soft–soft interaction with Au­(I), these results demonstrate that Lys is a potent nucleophile once the local environment—such as the hydrophobic pockets identified in Figure 4— facilitates the approach of the metal complex. While Lys residues are predominantly protonated at physiological pH, the proximity effect induced by the Npx anchor and the potential p*K*_a_ shifts within the protein hydrophobic microenvironments can facilitate the presence of the nucleophilic deprotonated form. The high intrinsic nucleophilicity of the Lys NZ (as quantified by the Fukui indices), combined with the electronic activation of AF-Npx (increased softness and electrophilicity, Table 3), provides a robust electronic rationale for the formation of stable Au-Lys bonds.

To fully evaluate the nature of these Au–N­(Lys) bonds across the experimentally observed geometric range (2.17–2.50 Å), a natural bond orbital (NBO) analysis was performed (Table S3). It reveals that while the interaction exhibits a significant distance-dependent polarization, a directional bonding pathway remains intact across the entire continuum. At the shorter coordination limit of 2.17 Å, the system fulfills all the criteria for a formal coordinate covalent bond, where the Au­(I) center holds a substantial 13.71% share of the electron density in a well-defined two-center bonding orbital (BD) driven by a directional hybrid orbital on the nitrogen headgroup (17.54% s-character). As the Au–N distance elongates to the long-range boundary of 2.49–2.50 Å, the covalent electron sharing is significantly reduced, with the polarization shifting heavily toward the nitrogen center (only 9.89% Au) and the nitrogen headgroup rehybridizing to an sp^6.72^ state. Importantly, even at the maximum distance of 2.50 Å, the NBO algorithm explicitly localizes a two-center BD rather than a decoupled lone pair. This mathematical assignment is reinforced by the absolute directional rigidity of the interaction, which maintains a strict orbital angular deviation of just 1.8° along the interatomic line of centers. These data demonstrate that the long-range Au-Lys interactions are best described as borderline coordinate covalent interactions captured at their ultimate geometric limit, rather than nonspecific electrostatic proximity effects.

In conclusion, the metalation of Lys residues in HEWL is not a random occurrence but the result of a precise synergy: the Npx moiety provides the spatial anchoring, while the enhanced electrophilicity of AF-Npx allows it to effectively exploit the intrinsic nucleophilicity of the Lys side chain.

## 3 Experimental Section

All solvents and reagents were purchased from Sigma-Aldrich (Darmstadt, Germany) and used without further purification. AF-Npx synthesis was carried out under nitrogen atmosphere using standard Schlenk techniques. The compound was stored at −20 °C in the dark. ^1^H, ^13^C­{^1^H}, and ^31^P­{^1^H} NMR spectra were recorded on a Bruker Avance II 400 spectrometer (^1^H 400.0 MHz, ^13^C 100.6 MHz, and ^31^P 162.0 MHz) with chemical shifts (δ, ppm) reported relative to the solvent peaks of the deuterated solvent. The spectra were processed using the MestreNova software (v12). Elemental analysis (C, H, and N) was accomplished with the VarioMICRO elemental analyzer. HR-ESI mass spectra were recorded using an Orbitrap high-resolution mass spectrometer (Thermo, San Jose, CA, USA), equipped with HESI source. Complex was solubilized in LC-grade anhydrous methanol, just before the analysis. Hen egg white lysozyme was purchased from Merck Life Science (Milan, Italy) and used without further purification.

### 3.1 Synthesis of AF-Npx

AF-Npx was prepared following our previously reported procedure.^26^ In brief, Et_3_PAuCl (80 mg, 0.23 mmol) was dissolved in EtOH (2 mL) in a Schlenk flask. [Ag]­[Npx] (78 mg, 0.23 mmol) was then added, and the reaction mixture was stirred magnetically for 2 h at room temperature, protected from light. After completion, the suspension was filtered through Celite, and the solvent was removed under reduced pressure. The resulting colorless oil was triturated with a small volume of Et_2_O for 1 h, affording a white solid. The product was dried under vacuum and collected. Yield: 100 mg (80%).

CHN: C_20_H_28_AuO_3_P·0.5H_2_O; required: C = 43.41; H = 5.28; N = 0.00. Found: C = 43.18; H = 5.38; N = 0.00. ^1^H NMR (400 MHz; CDCl_3_): 7.76 (s, 1H; H ar), 7.71 (m, 2H, H ar), 7.56 (dd, J = 8.4, 1.7 Hz, 1H, H ar), 7.11 (m, 2H, H ar), 3.92 (s, 3H, CH_3_–O), 3.86 (q, J = 7.1 Hz, 1H, CH), 1.80 (dq, J_HP_ = 18.3 Hz, J_HH_ = 7.6 Hz, 6H, CH_2_–P), 1.60 (d, J = 7.1 Hz, 3H, CH_3_), 1.17 (dt, J_HP_ = 18.8, J_HH_ 7.6 Hz, 9H, CH_3_ phosp.) (Figure S1). ^31^P­{^1^H}-NMR (162 MHz; CDCl_3_): 25.3 (Figure S2). ^13^C­{^1^H}-NMR (101 MHz; CDCl_3_): 180.2 (COO), 157.3 (C ar), 138.9 (C ar), 133.5 (C ar), 129.5 (C ar), 129.3 (C ar), 127.2 (C ar), 126.9 (C ar), 125.8 (C ar), 118.5 (C ar), 105.7­(C ar), 55.4 (CH_3_–O), 48.7 (CH), 19.8 (CH_3_), 17.9 (d; J_CP_ = 38.3 Hz; CH_2_–P), 9.1 (d; J_CP_ = 1.3 Hz CH_3_ phosp.) (Figure S3). HR-ESI-MS *m*/*z* [M–Na]+ = 567.13293 (theoretical: 567.13538); [M–K]^+^ = 583.10681 (theoretical: 583.10732) (Figure S4).

### 3.2 X-ray Crystallography

Crystals of metal-free HEWL were grown using the vapor diffusion method in the hanging drop variant. One μL of HEWL at a concentration of 13 mg mL^–1^ (0.9 mM) was mixed with 1 μL of the crystallization condition. Two different conditions were used: (i) 2.0 M sodium formate, 0.1 M Hepes buffer pH 7.5, (ii) 14% PEG 8K, 0.1 M sodium citrate and 0.1 M sodium cacodylate buffer pH 6.5. HEWL crystals grew within 1 day and were soaked with solutions containing the reservoir saturated with AF-Npx powder. After incubation, crystals were cryoprotected in solutions containing 70% reservoir and 30% glycerol and flash-cooled for X-ray diffraction analysis. Data collections were carried out on beamline ID23–2 of ESRF European Synchrotron, Grenoble, France, at a wavelength of 0.87 Å. Crystals were maintained at 100 K during the data collection. Data were collected on different crystals. The best crystals diffracted X-rays at 1.54 Å (crystal grown in condition (i), hereafter Structure **1)** and at 1.44 Å (crystal grown in condition (ii), hereafter Structure **2**). Data processing and scaling were performed using Global Phasing autoPROC pipelines.^47^ Data collection statistics are reported in Table S1. The structures were solved using the Fourier difference method by molecular replacement using Phaser^48^ and the structure of HEWL as starting model (PDB code:193L).^35^ Model building was carried out with Coot.^49^ The structures were isotropically refined with REFMAC5 in the CCP4 suite.^50,51^ Refinement statistics are reported in Table S1. Both structures were validated on PDB validation server and then deposited in the PDB under the accession codes29OHand29OL, respectively.

### 3.3 Computational Details

The solvent accessibility of the protein nucleophilic sites was assessed by calculating the solvent accessible surface area (SASA) on the metal-free protein (PDB code:193L)^35^ and on the metalated Structures **1** and **2**. Calculations were performed using the gmx sasa tool within the GROMACS package,^52^ employing a probe radius of 1.4 Å. The fractional exposure (%) for each residue was determined as the ratio between the SASA of the residue within the protein context and the SASA of the same residue in an isolated state. The apolar contribution to the SASA was estimated by summing the solvent-accessible areas of the side-chain carbon atoms. This approach allowed for a systematic classification of residues into moderately or highly exposed categories based on their initial exposure in the apo-form.

To support the elucidation of the metalation patterns detected in the Structure **1** and Structure **2**, the electronic structure of auranofin (AF) and its naproxen complex (AF-Npx) were comparatively investigated by means of quantum chemistry approaches.

Our calculations were performed within the framework of density functional theory (DFT), a well-established methodology for modeling the geometry and reactivity of transition metal complexes,^53–55^ including gold-based compounds.^56,57^ All calculations were conducted using the Gaussian 16 quantum chemistry package.^58^ The range-separated hybrid functional ωB97X-D^59^ was employed throughout, selected for its superior performance in describing molecular geometries, noncovalent interactions, and electronic energies.^60,61^ Geometries for all species were fully optimized in the aqueous phase using the def2TZVP basis set.^62,63^ Bulk solvent effects were accounted for via the Polarizable Continuum Model (PCM) in its Integral Equation Formalism (IEFPCM) variant,^64^ which has demonstrated high accuracy in predicting solvation free energies for both neutral and ionic species.^65,66^ Harmonic vibrational frequency analyses were performed at the same level of theory to confirm the nature of the stationary points and to derive zero-point energy (ZPE) and thermal corrections for the thermodynamic parameters.

To characterize the global electronic reactivity of the AF and AF-Npx complexes, conceptual DFT (cDFT) descriptors were calculated.^67,68^ The global chemical potential (μ), hardness (η), softness (*S*), and electrophilicity (ω), were determined from the energies of the highest occupied (*E*_HOMO_) and lowest unoccupied (*E*_LUMO_) molecular orbitals at the ωB97X-D/def2TZVP level. These parameters are reported in hartrees (Ha), with the exception of softness (*S*), which is in Ha^1–^. These parameters were calculated according to the following finite difference approximations: $\mu \approx \frac{1}{2}\left(E_{\mathrm{HOMO}}+E_{\mathrm{LUMO}}\right)$ ; $\eta \approx \frac{1}{2}\left(E_{\mathrm{LUMO}}-E_{\mathrm{HOMO}}\right)$ ; $S=\frac{1}{2\eta }$ ; and $\omega =\frac{\mu^{2}}{2\eta }$ . All global descriptors are reported in hartrees (Ha), except for softness (S), reported in Ha^1–^.

The local reactivity of the protein binding sites was quantified using dimensionless nucleophilic Fukui indices (*f*_k_^–^).^69^ These indices were derived from Mulliken population analysis for the target nitrogen atoms of the His and Lys residues (*N*_ϵ_ for Lys; *N*_δ1_ and *N*_δ2_ for His) using the optimized geometries at the ωB97X-D/def2TZVP level in water. The indices were calculated as *f*_k_^–^ = *q_k_* (N) – *q*_k_(*N* – 1), where *q*_*k*_(*N*) and *q*_*k*_(*N* – 1) represent the atomic charges of the neutral and cationic species, respectively. This approach provided a localized numerical assessment of the intrinsic nucleophilicity of the competing nitrogen centers involved in the metalation process.

To further investigate the nature and electronic parameters of the localized Au–N­(Lys) coordination bonds across the experimentally observed geometric continuum, NBO analysis was performed.^70^ Indeed, NBO analysis represents an exceptionally powerful tool for evaluating donor–acceptor interactions and hybridization changes.^71,72^ We used it to calculate the localized two-center bond orbital occupancies, percentage polarization fractions, nitrogen headgroup hybridizations, and orbital angular deviations from the line of centers to clarify the continuous transition from a formal coordinate covalent bond to a borderline dative/electrostatic interaction at the 2.50 Å threshold.

## 4 Conclusions

The interaction between the AF derivative AF-Npx and the model protein HEWL was investigated through a combination of X-ray crystallography and computational analysis. Structural studies revealed that the complex undergoes activation via ligand dissociation upon exposure to the protein, releasing Au­(I) fragments that coordinate to nucleophilic sites. In addition to the canonical His15 metalation site commonly observed in the structure of the adducts of Au complexes with HEWL, unprecedented binding events involving Lys96, Lys116, and Lys13 were identified.

Computational analysis provided comprehensive mechanistic interpretation. Solvent accessibility calculations, using the apo protein as a baseline, and hydrophobic cluster mapping highlighted a dual-recognition mode: while highly exposed lysine residues are metalated through simple kinetic accessibility, the metalation of less accessible residues, such as Lys96, is directed by the Npx moiety acting as a thermodynamic anchor. Furthermore, DFT calculations revealed that replacing the thiosugar with Npx fundamentally reconfigures the electronic properties of the complex. Indeed, the AF-Npx derivative displays increased global electrophilicity and a lower electronic chemical potential compared to AF. While the complex also exhibits an increased global softness, the enhanced electrophilicity acts as the primary driving force to overcome the canonical HSAB mismatch, providing the necessary electronic activation to target and form stable Au-(N)­Lys bonds, which had not been previously observed for Au­(I) compounds interacting with lysozyme. These observations validate previous predictions suggesting that the precovalent phase is not a random anchoring process, but a crucial preparatory step governed by the electronic redistribution induced by the ligand.^32^

Overall, these findings demonstrate that ligand substitution can profoundly influence both the reactivity and the protein-targeting behavior of Au­(I) metallodrugs, effectively expanding their canonical reactivity. Lysine metalation by Au compounds is particularly noteworthy since lysines are among the most abundant and functionally relevant protein surface residues. Redirecting Au­(I) reactivity toward these residues may expand the range of accessible protein targets and contribute to differences in biological activity, biodistribution, or toxicity compared to conventional Au­(I) compounds. Although further studies are required, especially for a deeper biological evaluation, these findings suggest that ligand engineering may offer a viable strategy to tune residue selectivity and protein recognition by Au-based drugs.^73,74^

By modulating both the spatial recognition and electronic reactivity, the Npx ligand directs the metal fragment toward specific protein microenvironments that are otherwise inaccessible. This anchor-and-activate strategy provides a significant contribution to the deeper understanding of metallodrug–protein recognition and possibly offers a robust blueprint for the design of next-generation Au-based therapeutics with different site-selectivity.

## Supplementary Material



## Data Availability

The procedures and data supporting this article have been included as part of Supporting Information. Further data underlying this article will be shared on reasonable request to the corresponding authors.

## Notes

1H; ^13^C, and ^31^P NMR spectra and ESI-MS spectrum of AF-Npx complex (Figures S1–S4); data collection statistics for the HEWL:AF-Npx adducts (Table S1); X-ray structures formed upon reaction of HEWL with Au compounds deposited in the PDB (Table S2); NBO parameters for the Au–N coordination continuum observed in the crystallized structures (Table S3) (PDF)
