# Supplementary material for: Tuning Au Reactivity Beyond Canonical Targets: Ligand-Driven Au(I) Metalation of Lysine Residues in Hen Egg White Lysozyme
Source: Inorg Chem. 2026 Jul 8;65(28):16296–304. doi: 10.1021/acs.inorgchem.6c01884 (PMC13390033; doi:10.1021/acs.inorgchem.6c01884)
Supplement: Supplementary file 1 [file ic6c01884_si_001.pdf]

Supporting Information

Tuning Au reactivity beyond canonical targets: Ligand-driven Au(I) metalation of lysine residues in hen egg white lysozyme

Davide Piroddu,[a‡] Luca Famlonga,[b‡] Iogann Tolbatov,[a] Giarita Ferraro,[c]* Lorenzo Chiaverini,[b]* Antonello Merlino,[c] Diego La Mendola,[b] Alessandro Marrone,[d] and Tiziano Marzo[b]*

[a]*Department of Chemical, Physical, Mathematical and Natural Sciences, University of Sassari, Via Vienna 2, 07100 Sassari, Italy.*

[b]*Department of Pharmacy, University of Pisa, Via Bonanno Pisano 6, 56126, Pisa, Italy.*

[c]*Department of Chemical Sciences, University of Naples Federico II, Via Cintia 21, I-80126 Napoli, Italy.*

[d]*Department of Pharmacy, University of Chieti-Pescara "G. D'Annunzio", Viale Pindaro 42, 66100 Chieti, Italy.*

‡ Equally contributed

Corresponding Authors

*Iogann Tolbatov, Department of Chemical, Physical, Mathematical and Natural Sciences, University of Sassari, Via Vienna 2, 07100 Sassari, Italy; **tolbatov.i@gmail.com**;

*Lorenzo Chiaverini, Department of Pharmacy, University of Pisa, Via Bonanno Pisano 6, 56126, Pisa, Italy. **lorenzo.chiaverini@phd.unipi.it**

*Giarita Ferraro, Department of Chemical Sciences, University of Naples Federico II, Via Cintia 21, I-80126 Napoli, Italy. **giarita.ferraro@unina.it**

Table of Content

1.1 ^{1}H-NMR Spectrum 3

1.2 ^{31}P-NMR Spectrum 4

1.3 ^{13}C-NMR Spectrum 5

1.4 HR-ESI mass spectrum 6

2.1 Table S1 7

2.2 Table S2 8

2.3 Table S3. 10

3 References 11

1.1 ¹H-NMR Spectrum

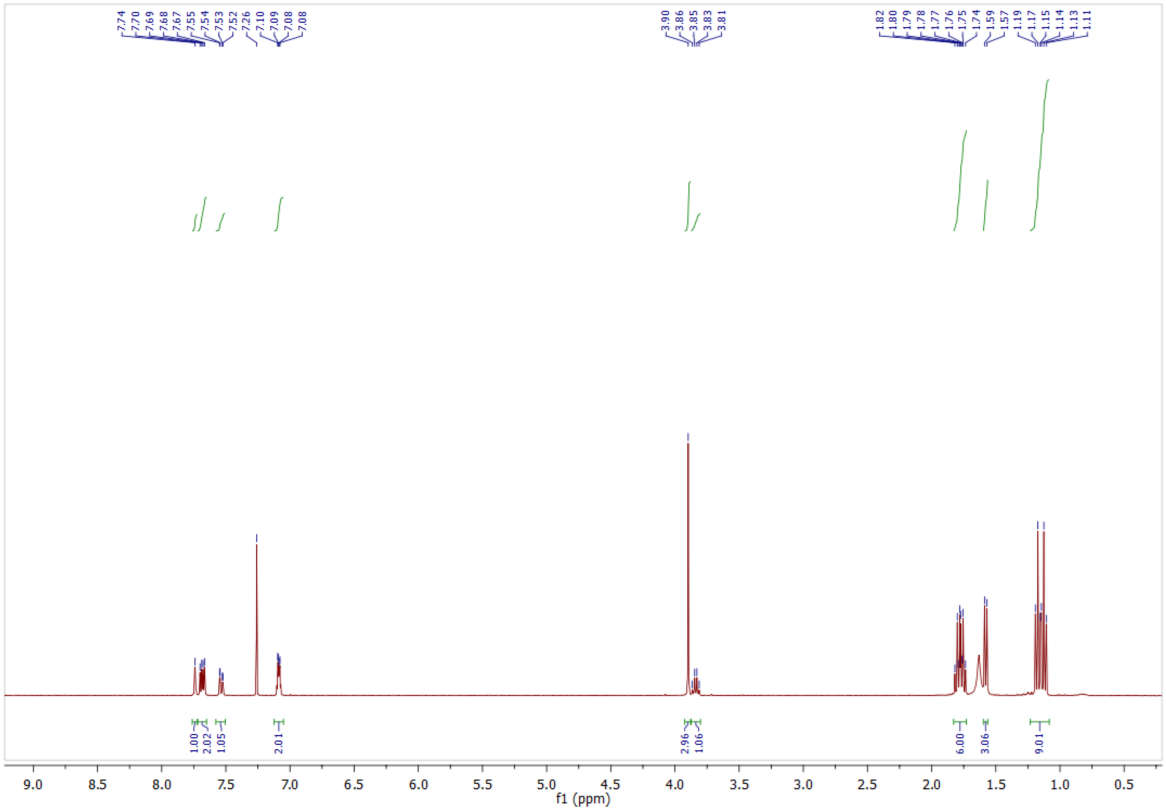

Figure S1. ¹H-NMR (400MHz; $CDCl_3$) spectrum of **AF-Npx**.

1.2 ^{31}P-NMR Spectrum

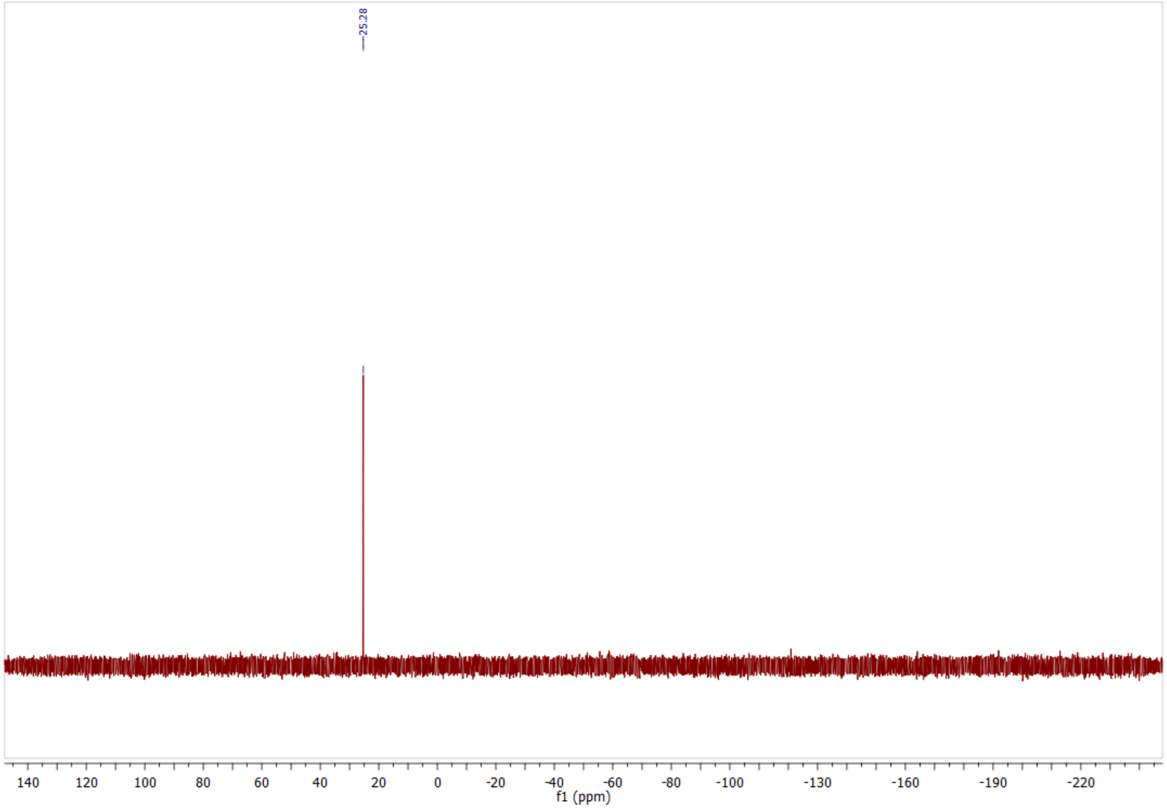

Figure S2. ^{31}P-NMR (160MHz; $CDCl_3$) spectrum of **AF-Npx**.

1.3 ¹³C-NMR Spectrum

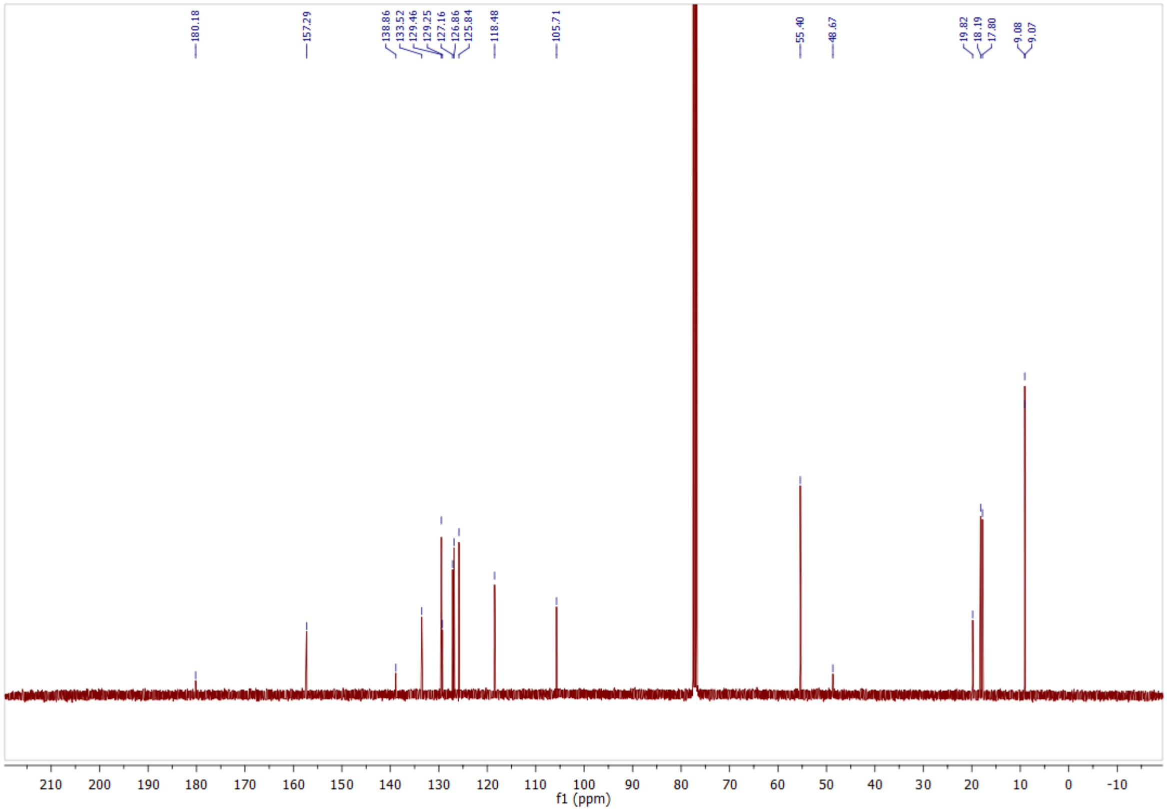

Figure S3. ^{13}C-NMR (101MHz; $CDCl_3$) spectrum of **AF-Npx**.

1.4 HR-ESI mass spectrum

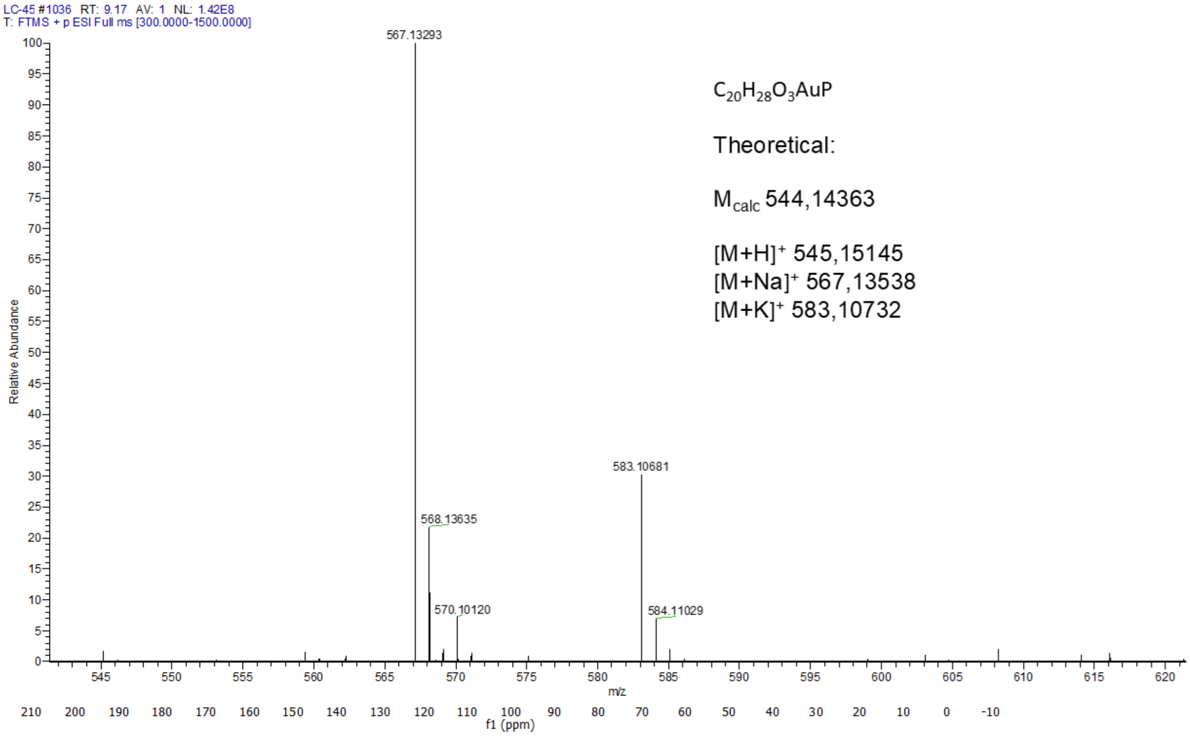

Figure S4. HR-ESI mass spectrum of **AF-Npx.**

2.1 Table S1

Table S1. Data collection and refinement statistics.

	*Structure **1***	*Structure **2***
PDB code	29OH	29OL
Crystallization condition	2.0 M sodium formate, 0.1 M Hepes buffer pH 7.5	14% PEG 8K, 0.1 M sodium citrate and 0.1 M sodium cacodylate buffer pH 6.5
Data collection		
Space group	$P4_12_12_1$	$P4_12_12$
a (Å)	79.394	78.660
b (Å)	79.394	78.660
c (Å)	35.815	37.327
α/β/γ (°)	90.0/90.0/90.0	90.0/90.0/90.0
Resolution range (Å)	32.65 – 1.54 (1.64 – 1.54)	39.33 – 1.44 (1.51 – 1.44)
Observations	238805 (12260)	445047 (21848)
Unique reflections	15114 (756)	20064 (1003)
Completeness (%)	92.9 (42.9)	94.5 (46.7)
Redundancy	15.8 (16.2)	22.2 (21.8)
Rmerge (%)	0.204 (3.965)	0.202 (3.696)
Average I/σ(I)	7.9 (1.4)	12.9 (1.0)
$CC_{1/2}$	0.995 (0.633)	0.994 (0.627)
Anom. completeness (%)	93.2 (44.3)	94.4 (47.7)
Anom. Multiplicity	8.6 (8.4)	11.9 (11.3)
Refinement		
Resolution (Å)	32.65 - 1.54	39.33 – 1.44
Reflections	14315	19112
R-factor/R_{free}	0.208/0.250	0.181/0.200
Non-H atoms in the refinement	1106	1106
Overall B-factor (Å^2)	23.57	20.47
R.m.s.d. bonds (Å)	0.008	0.012
R.m.s.d. angles (°)	1.630	1.812
Estimated occupancy of Au-(His15)	0.70	0.50/0.20
Estimated occupancy of Au-(Lys13)	-	0.20
Estimated occupancy of Au-(Lys96)	0.50	0.20
Estimated occupancy of Au-(Lys116)	0.20	0.30
B-factor of Au-(His15) (Å^2)	27.83	26.56/49.59
B-factor of Au-(Lys13) (Å^2)	-	40.96
B-factor of Au-(Lys96) (Å^2)	68.10	38.33
B-factor of Au-(Lys116) (Å^2)	37.76	42.29
Distance (Å) Au-ND1/ Au-NE2 (His15)	2.10	2.01/2.21
Distance (Å) Au-NZ (Lys13)	-	2.17
Distance (Å) Au-NZ (Lys96)	2.35	2.29
Distance (Å) Au-NZ (Lys116)	2.49	2.50
Ramachandran plot		
Most favoured/Additional allowed	116 (93.55%)	118 (95.16%)
Outliers	0 (0.00%)	1 (0.81%)

2.2 Table S2

Table S2. X-ray structures of the adducts formed upon reaction of HEWL with gold compounds reported in the PDB.

PDB code	Metal complex	Binding site	Au-containing fragment bound to HEWL	Distance (Å)	Occupancy	Ref.
4OOT	Aubipy[c] [(bipy^{dmb}-H)Au(OH)][PF_6] (where bipy^{dmb}-H = deprotonated 6-(1,1-dimethylbenzyl)-2,2′-bipyridine)	NE2 Gln121B	Au(I) ion (coordinated to a water molecule)	2.38	0.60	1
4ZFP	$AuSac_2$ [NH_4][Au(Saccarinate)$_2$]	NE2 His15	Au(I) ion (coordinated to a nitrate ion)	2.10	0.85	2
		ND1 His15	Au(I) ion	1.92	0.35	
		SD Met105	Au(I) ion (coordinated to a water molecule)	2.43	0.50	
4LFP	$AuSac_2$ [NH_4][Au(Saccarinate)$_2$]	NE2 His15	Au(I) ion (coordinated to a chloride ion)	2.27	0.60	
4LFX	Auoxo6 [(bipy^{2Me})$_2$Au$_2$(μ-O)$_2$][PF_6]$_2$ (where bipy^{2Me} = 6,6′-dimethyl-2,2′-bipyridine)	NE2 His15	Au(I) ion (coordinated to a chloride ion)	2.28	0.50	3
4LGK	Au_2phen [(phen^{2Me})$_2$Au$_2$(μ-O)$_2$][PF_6]$_2$ (where phen^{2Me} = 2,9-dimethyl-1,10-phenanthroline)	NE2 His15	Au(I) ion (coordinated to a chloride ion)	2.84	0.40	
4QY9	Auoxo3 [Au$_2$(bipy^{Me})$_2$(μ-O)$_2$][PF_6]$_2$ (where bipy^{Me} = 6-methyl-2,2′-bipyridine)	NE2 His15	Au(I) ion (coordinated to a water molecule)	2.33	0.40	4
		ND1 His15	Au(I) ion	2.37	0.40	
6SEU	Compound **2** Au(propyl-sulfonate) (*N*,*N*-pyridylbenzimidazole)	ND1 His15/ ND2 Asn93<br>ND1 His15	Au(I) ions (alternative positions)	2.31/2.83<br>2.69	0.40/0.25	5
		NE2 His15	Au(I) ion (coordinated to a water molecule)	2.49	0.40	
		SD Met105	Au(I) ion	3.38	0.50	

6SEW	Compound **2**<br>Au(propyl-sulfonate)<br>(*N*,*N*-pyridylbenzimidazole)	ND1 His15/<br>ND2 Asn93	Au(I) ion	2.27/2.95	0.50	
6SEX	Compound **1**<br>Au(ethyl) (*N*,*N*-pyridylbenzimidazole)	ND1 His15/<br>OD1 Asn93<br><br>ND1 His15	Au(I) ions<br>(alternative<br>positions)	2.29/2.71<br><br>2.68	0.50/0.40	
		NE2 His15	Au(I) ion<br>(coordinated to a<br>water molecule)	2.14	0.30	
		SD Met105	Au(I) ion<br>(coordinated to a<br>water molecule)	2.51	0.25	
6SEZ	Compound **1**<br>Au(ethyl) (*N*,*N*-pyridylbenzimidazole)	ND1 His15/<br>OD1 Asn93	Au(I) ion	2.61/2.87	0.40	
		NE2 His15	Au(I) ion<br>(coordinated to a<br>water molecule)	2.50	0.25	
		SD Met105	Au(I) ion	3.27	0.30	
6SET	Compound **1**<br>Au(ethyl) (*N*,*N*-pyridylbenzimidazole)	OD1 Asn19*<br><br>O Gly22	Au(I) ions<br>(alternative<br>positions)<br><br>Au(I) ion	3.13/2.80<br><br>3.00	0.35<br><br>0.15	
		ND1 Asn19/<br>OD1 Asn44*	Au(I) ions<br>(alternative<br>positions)	2.28/2.74/2.90<br>3.04	0.20<br><br>0.30	
		O Tyr118	Au(I) ion	3.00	0.30	
7R1Q	**Au2**<br>[Au(NHC-glucoside)Br]	ND1 His15	Au(I) ion	2.02	0.20	6
		N Lys 1	Au(I) ion<br>(coordinated to a<br>water molecule)	2.25	0.20	
3P4Z	$ClAuS(CH_2CH_2OH)_2$	NE2 His15	Au(I) ion<br>(coordinated to a<br>chloride ion)	2.01	1.00	7,8
3P64	$ClAuS(CH_2CH_2OH)_2$	NE2 His15	Au(I) ion<br>(coordinated to a<br>chloride ion)	2.01	0.50	

3P65	$ClAuS(CH_2CH_2OH)_2$	NE2 His15	Au(I) ion (coordinated to a chloride ion)	1.95	0.30	

2.3 Table S3.

Table S3. NBO parameters for the Au-N coordination continuum observed in the crystallized structures. Electronic properties, including localized two-center bond orbital occupancy, percentage polarization fractions on the N and Au atoms, N headgroup hybridization, and orbital angular deviation from the interatomic line of centers, are reported as a function of the optimized Au-N distance to characterize the transition from a formal coordinate covalent bond to a borderline dative/electrostatic interaction at the 2.50 Å limit.

Distance (Å)	Occupancy (e)	% Polarization		N hybridization	Deviation (°)
		N	Au		
2.17	1.98	86.29	13.71	$sp^{4.70}$ (17.54% s)	1.7
2.29	1.978	87.8	12.2	$sp^{5.43}$ (15.56% s)	1.8
2.35	1.976	88.51	11.49	$sp^{5.80}$ (14.70% s)	1.8
2.49	1.973	90.02	9.98	$sp^{6.66}$ (13.05% s)	1.8
2.5	1.973	90.11	9.89	$sp^{6.72}$ (12.95% s)	1.8

3 References

(1) Messori, L.; Cinellu, M. A.; Merlino, A. Protein Recognition of Gold-Based Drugs: 3D Structure of the Complex Formed When Lysozyme Reacts with Aubipyc. *ACS Med. Chem. Lett.* **2014**, *5* (10), 1110–1113. https://doi.org/10.1021/ml500231b.

(2) Ferraro, G.; Massai, L.; Messori, L.; Cinellu, M. A.; Merlino, A. Structural Evidences for a Secondary Gold Binding Site in the Hydrophobic Box of Lysozyme. *Biometals* **2015**, *28* (4), 745–754. https://doi.org/10.1007/s10534-015-9863-7.

(3) Messori, L.; Scaletti, F.; Massai, L.; Cinellu, M. A.; Gabbiani, C.; Vergara, A.; Merlino, A. The Mode of Action of Anticancer Gold-Based Drugs: A Structural Perspective. *Chem. Commun.* **2013**, *49* (86), 10100–10102. https://doi.org/10.1039/C3CC46400H.

(4) Russo Krauss, I.; Messori, L.; Cinellu, M. A.; Marasco, D.; Sirignano, R.; Merlino, A. Interactions of Gold-Based Drugs with Proteins: The Structure and Stability of the Adduct Formed in the Reaction between Lysozyme and the Cytotoxic Gold(III) Compound Auoxo3. *Dalton Trans.* **2014**, *43* (46), 17483–17488. https://doi.org/10.1039/C4DT02332C.

(5) Ferraro, G.; Giorgio, A.; Mansour, A. M.; Merlino, A. Protein-Mediated Disproportionation of Au(I): Insights from the Structures of Adducts of Au(III) Compounds Bearing N,N-Pyridylbenzimidazole Derivatives with Lysozyme. *Dalton Trans.* **2019**, *48* (37), 14027–14035. https://doi.org/10.1039/C9DT02729G.

(6) Annunziata, A.; Ferraro, G.; Cucciolito, M. E.; Imbimbo, P.; Tuzi, A.; Monti, D. M.; Merlino, A.; Ruffo, F. Halo Complexes of Gold(I) Containing Glycoconjugate Carbene Ligands: Synthesis, Characterization, Cytotoxicity and Interaction with Proteins and DNA Model Systems. *Dalton Trans.* **2022**, *51* (27), 10475–10485. https://doi.org/10.1039/D2DT00423B.

(7) Wei, H.; Wang, Z.; Zhang, J.; House, S.; Gao, Y.-G.; Yang, L.; Robinson, H.; Tan, L. H.; Xing, H.; Hou, C.; Robertson, I. M.; Zuo, J.-M.; Lu, Y. Time-Dependent, Protein-Directed Growth of Gold Nanoparticles within a Single Crystal of Lysozyme. *Nature Nanotech* **2011**, *6* (2), 93–97. https://doi.org/10.1038/nnano.2010.280.

(8) Merlino, A.; Caterino, M.; Russo Krauss, I.; Vergara, A. Missing Gold Atoms in Lysozyme Crystals Used to Grow Gold Nanoparticles. *Nature Nanotech* **2015**, *10* (4), 285–285. https://doi.org/10.1038/nnano.2015.53.
